# Multirate ECG Processing and k-Nearest Neighbor Classifier Based Efficient Arrhythmia Diagnosis

**DOI:** 10.1007/978-3-030-51517-1_29

**Published:** 2020-05-31

**Authors:** Saeed Mian Qaisar, Moez Krichen, Fatma Jallouli

**Affiliations:** 8 Digital Research Centre of Sfax, Sfax, Tunisia; 9 Institut Mines-Télécom, CNRS, Paris, France; 10 Université de Sherbrooke, Sherbrooke, QC Canada; 11 Digital Research Centre of Sfax, Sfax, Tunisia; 12 University of Sfax, Sfax, Tunisia; 13 College of Engineering, Effat University, Jeddah, Kingdom of Saudi Arabia; 14 Faculty of CSIT, Al-Baha University, Al Bahah, Saudi Arabia; 15 ReDCAD Laboratory, University of Sfax, Sfax, Tunisia; 16 Faculty of Medicine of Sfax, Sfax, Tunisia

**Keywords:** Multirate processing, ECG, Arrhythmia, Wavelet, Features extraction, Classification

## Abstract

The goal of this work is to make a contribution to the development of computationally efficient multirate Electrocardiogram (ECG) automated detectors of arrhythmia. It utilizes an intelligent combination of multirate denoising plus wavelet decomposition for an effective realization of the ECG wireless implants. The decomposed signal subband features are mined and in next step these are utilized by the mature k-Nearest Neighbor (KNN) classifier for arrhythmia diagnosis. The multirate nature substantially reduces the processing activity of the system and thus allows a dramatic decrease in energy consumption compared to traditional counterparts. The performance of the system is estimated also in terms of the classification performance. Obtained results reveal an overall 22.5-fold compression gain and 4-folds processing outperformance over the traditional equals while securing 93.2% highest classification accuracy and specificity of 0.956. Findings confirm that the proposed solution could potentially be embedded in contemporary automatic and mobile cardiac diseases diagnosis systems.

## Introduction

Cardiovascular diseases have drawn global attention. This is due to its increasing prevalence and incidence [1, 14]. Electrocardiogram (ECG) measures electrical activities with respect to time. Manual examination of cardiac arrhythmias can be time consuming and complicated. This challenge may be solved using computer-aided automatic cardiac decision tools. The computer-aided or pattern-based recognition systems could increase the effectiveness of cardiac health analysis by detecting subtle differences in frequency and amplitude components of the heartbeat [2].

Many scientists have previously explored computer-assisted solutions for cardiac health monitoring as reviewed in [7]. Preprocessing is the first ECG processing stage. The popular ECG denoising methods are the finite impulse response (FIR) filtering, principle component analysis (PCA) and Kalman filtering [2, 8]. The extraction of features is one of the essential steps of computer-aided ECG diagnostic solutions. Certain extensively used ECG signal feature extraction approaches are the “Wavelet Transform” (WT), “Discrete Cosine Transform” (DCT) and “Short Time Fourier Transform” (STFT). The pertinent signal features are afterward employed for the classification purpose. Techniques adopted for this purpose are the “Naïve Bias”, the “K-Nearest Neighbor” (KNN), the “Artificial Neural Networks” (ANN) and the “Support Vector Machine” (SVM).

Classical ECG systems are by definition time-invariant [3, 4]. This can lead to inefficient use of system resources and energy consumption [2, 5]. For such signals, an effective solution can be achieved by diminishing the rates of data collection, processing and transmission [5]. In this framework, multirate signal processing tactics have been employed [6]. The subsampling is intelligently employed in the suggested framework. It allows overcoming the downsides of the counter fix rate ECG processing approaches [3, 4]. Therefore, it allows realizing a simplified and power efficient ECG wireless implant with a real-time compression of data.

## Materials and Methods

**Fig. 1.**
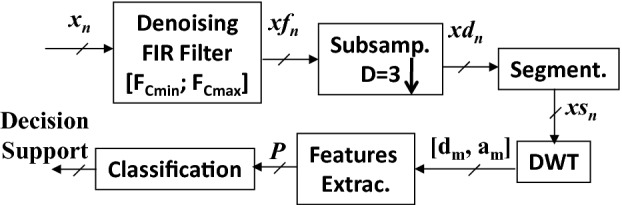
Block diagram of the adopted system

Figure 1 illustrates the adopted system block level diagram. A description of the different modules of the system is given in the coming subsections.

### Dataset

In this study, the ECG signals, obtained from a standard ECG dataset are used [1]. 3 different ECG classes the “Wolff-Parkinson-White” (WPW), “Right Bundle Branch Block” (RBBB) and the “Normal Sinus Rhythm” (N) are considered. ECG analog signals are band limited up to 60 Hz and each channel is recorded via an 11-Bit resolution analog to digital converter (ADC). The employed sampling frequency is of 360 Hz. The digitized versions of intended ECG signals are splitted into fixed length segments to split the continuous time signals into ECG impulses. Each impulse is considered as an instance. In order to avoid any biasing an equal representation is selected for each considered class. In this framework, 150 instances are considered for each class. It results in total 450 instances from 3 ECG classes.

### Denoising

The digitized signal $x_{n}$ is denoised by using an offline designed band-pass FIR filter. The denoising diminishes the noise like the “Power Line Interference” (PLI) and “Baseline Wander” (BW) from the ECG signal. It improves the efficiency of collection and classification of the features. The ECG signal’s useful frequency range lies between [0.5; 50] Hz [9, 10]. Accordingly, a band-pass linear phase filter is configured offline for the cut-off frequencies of [${\text{Fc}}_{\text{L}}$ = 0.5; ${\text{Fc}}_{\text{H}}$ = 50] Hz it resulted in a 122^nd^ order filter designed for $F_{S} = 360$ Hz. For proper filtering, ${\text{Fc}}_{\text{H}}$ is kept less than half of the signal sampling rate [5]. Therefore, $F_{S} = 360$ Hz fulfils this criterion.

### Subsampling

The functioning of conventional ECG acquisition and analysis processes is of time-invariant nature [2–4]. Consequently, a worst-case parameterization is enforced [5]. It causes the processing ineffectiveness in the case of time-varying and sporadic ECG signals. These inadequacies can be diminished by using multirate processing approaches [2, 5, 6]. In this framework, the denoised signal $xf_{n}$ is subsampled with a factor of $D = 4$ to obtain $xd_{n} = xf_{Dn}$. Subsampling without a prior digital antialiasing filtering can cause aliasing [6]. However, a proper choice of $D$ allows to perform subsampling without prior filtering. In this case, the selected value of $D$ should respect the condition: $D \le \frac{{F_{S} }}{{F_{Nyq} }} = 3.6$. Here, $F_{S} = 360$ Hz, $F_{Nyq} = 2.\,f_{max}$ and $f_{max}$ is the bandwidth of $xf_{n}$ and is equal to ${\text{Fc}}_{\text{H}}$ = 50 Hz. It shows that for the chosen $D = 3$ subsampling does not cause aliasing.

### Segmentation

In order to split the continuous time ECG records into ECG pulses, $xd_{n}$ is divided in 0.9-s length segments. Each segment, $xs_{n}$, contains one ECG pulse. The segmentation is realized by using fixed length rectangular windows [6]. The process can be mathematically depicted as:

$$ ys_{n} = \sum\nolimits_{{n = \tau - \frac{{L_{T} }}{2}}}^{{\tau + \frac{{L_{T} }}{2}}} {yd_{n} w_{n - \tau } .} $$

Here, $L_{T}$ and $\tau$ are respectively the length in seconds and the central time of an intended segment.

### Discrete Wavelet Transform

The “Wavelet Transform” (WT) can be mathematically expressed by Eq. (1) where, s and u respectively represent the dilation and the translation parameters.

1 $$ W_{x}^{\psi } \left( {u,s} \right) = \frac{1}{\sqrt S }\int_{ - \infty }^{ + \infty } {x(t)\psi * \left( {\frac{(t - u)}{s}} \right)dt} . $$

A discrete time wavelet transform (DWT) is used for decomposing the $xs_{n}$. A translation-dilation representation is attained by employing digital filters. In this case, each segment $xs_{n}$ is decomposed through the “Daubechies Algorithm” based wavelet decomposition process. It consists of half-band low-pass filter and high-pass filter with subsampling with a factor of two. It allows the computation of approximation, $a_{m}$ and detail, $d_{m}$, coefficients at each level of decomposition.

The mathematical processes of computing $a_{m}$ and $d_{m}$ are respectively depicted by Eq. (2) and Eq. (3). Where, $m$ represents the level of decomposition. In this study a third level of decomposition is employed. Therefore, $m \in \left\{ {1, 2,3} \right\}$. $g_{2n - k}$ and $h_{2n - k}$ are respectively the half-band low-pass and high-pass filters using a subsampling factor of two.

2 $$ \varvec{a}_{\varvec{m}} = \sum\nolimits_{{\varvec{k} = 1}}^{{\varvec{K}_{\varvec{g}} }} {\varvec{ys}_{\varvec{n}} \varvec{.}\,\varvec{g}_{{2\varvec{n} - \varvec{k}}} } . $$

3 $$ \varvec{d}_{\varvec{m}} = \sum\nolimits_{{\varvec{k} = 1}}^{{\varvec{K}_{\varvec{g}} }} {\varvec{ys}_{\varvec{n}} \varvec{.}\,\varvec{h}_{{2\varvec{n} - \varvec{k}}} } . $$

### Features Extraction

The wavelet coefficients, obtained for each intended subband, $d_{1} = [60, 120]$ Hz, $d_{2} = [30, 60]$ Hz, $d_{3} = [15, 30]$ Hz and $a_{3} = [0, 15]$ Hz are used for mining the discriminative and classifiable features. 4 statistical features are extracted from each subband. These are described in the following.

***Energy (E)*** is calculated by adding all the absolute values of subband coefficients. ***Kurtosis of the signal (K)*** is a measure of the curvature of the considered subband coefficients. ***Peak positive value (PV)*** is the maximum positive value of the intended subband coefficients. ***Peak negative value (NV)*** is the maximum negative value of the intended subband coefficients.

### Classification

After features extraction, each instance is presented in the form of a reduced data matrix, composed of 16 features. The intended dataset is composed of 3 ECG classes namely the “Normal Sinus Rhythm (N), the “Right Bundle Branch Block” (RBBB) and the “Wolff-Parkinson-White” (WPW). For equal representation, 150 instances are taken into consideration for every class. Thus, in total 450 ECG instances are considered. After features extraction, the resulting data matrix has a size of 450 × 16. To classify this data matrix, the “k-Nearest Neighbor” (KNN) classification algorithm is employed.

The KNN is well known for its ability of delivering high quality results even for applications wit high complexity [16]. In a data set, the features’ distance is used by KNN to decide which data belongs to what class. When the distance in the data is near, a group is formed, and when the distance in the data is far, other groups are formed. A category membership might be the output of the KNN classifier. The categorization of an object is done through the majority vote by its neighbors. That is, the object is added to the class which is most common among its *k* closest neighbors (*k* could generally be a small positive whole number). The object is assigned solely to the nearest neighbor’s single classification if the *k* equals one [11].

### Evaluation Measures

**Compression Ratio** compares the designed system performance in terms of reduction in the amount of information to be classified compared to the conventional approach where acquired ECG data points are transmitted towards classifier without performing any features selection. If $\varvec{N}_{\varvec{r}}$ and $\varvec{P}$ are respectively the count of data points to be classified, for a given time length of $\varvec{L}_{\varvec{T}}$-Sec., in the conventional and the devised approach then the compression ration, $\varvec{R}_{{\varvec{COMP}}}$, can be calculated as:

$$ R_{COMP} = \frac{{N_{r} }}{P}. $$

**Fig. 2.**
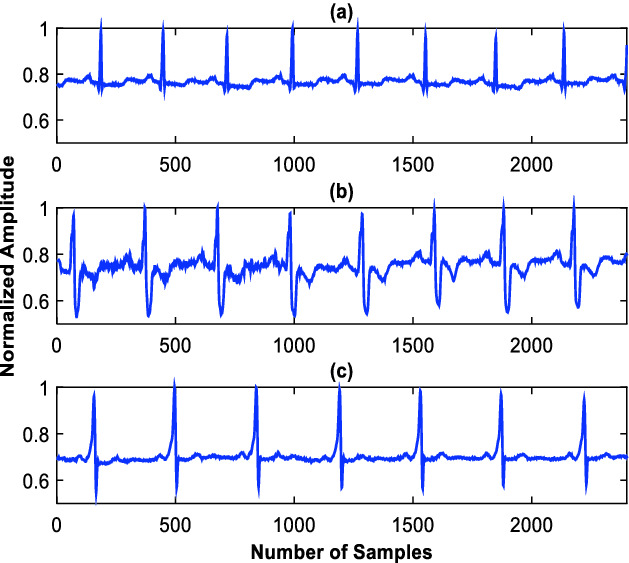
Examples of the ECG signals. (a) (N), (b) (RBBB) and (c) (WPW).

**Computational Complexity** compares the designed system performance with the fixed-rate counter equals in terms of the count of required standard operations like additions, multiplications and divisions [12]. In conventional case, the denoised signal is segmented by employing a rectangular window. It splits the incoming samples sequence in $\varvec{L}_{\varvec{T}}$-Sec. segments. Each segment is composed of $\varvec{N}_{\varvec{r}}$ samples. The processing cost of this process is negligible compared to operations like additions and multiplications [12]. Each segment is further split into subbands by using the 3^rd^ level Daubechies wavelet decomposition. It consists of half-band FIR high-pass and low-pass filters with a subsampling factor of two. Let $\varvec{Kg}$ be the order of half-band filters and same filters are employed at all levels of decomposition. It is well known that a $\varvec{Kg}$ order filter performs $\varvec{Kg }$ additions and $\varvec{Kg}$ multiplications [5]. Therefore, the computational complexity of this fixed rate wavelet decomposition process $\varvec{C}_{{\varvec{FR} - \varvec{WD}}}$ can be mathematically expressed by Eq. (4). This mathematical derivation is also clear from Fig. 2.

4 $$ C_{FR - WD} = \underbrace {{3.5 \times Kg.\,N_{r} }}_{Additions} + \underbrace {{3.5 \times Kg.\,N_{r} }}_{Multiplications}.$$

For the case of designed solution $xf_{n}$ is firstly subsampled and then $xd_{n}$ is segmented by employing a rectangular window. Each segment is composed of $N = 0.25 \times N_{r}$ samples. If $Kg$ is the order of half-band filters and same filters are employed at all levels of decomposition then the computational complexity of this process $C_{P - WD}$ can be mathematically expressed by Eq. (5). If $M = 0.5 \times M_{r}$ is the count of samples processed by the denoising module then the total computational complexity for the designed front-end processing chain can be expressed by using Eq. (5).

5 $$ C_{P - WD} = \underbrace {{0.875 \times Kg.\,N_{r} }}_{Additions} + \underbrace {{0.875 \times Kg.\,N_{r} }}_{Multiplications} $$

**Classification Accuracy and Specificity** are used to evaluate the overall system precision. The processes may be formally described by means of Eq. (6) and Eq. (7). Where, “True Positives” (TP) and “True Negatives” (TN) are correct classifications. “False Negatives” and ”False Positives” (FP) (FN) are wrong classification results [13].

6 $$ Accuracy = \frac{{T_{P} + T_{N} }}{{T_{P} + T_{N} + F_{P} + F_{N} }} \times 100\%.$$

7 $$ Specificity = \frac{T_{N}}{T_{N}  + F_{P}}. $$

## Results and Discussions

**Fig. 3.**
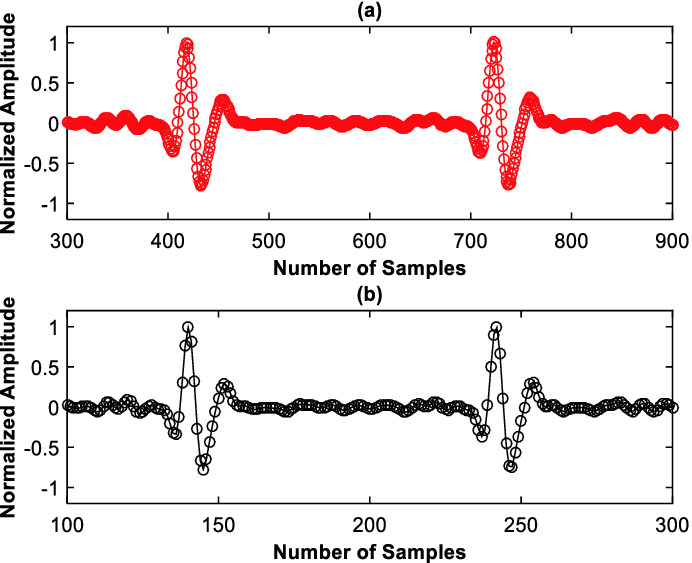
Example of denoised RBBB signal (a) and example of decimated RBBB signal (b).

Examples of the considered ECG signal classes are shown in Fig. 2. These incoming signals $x_{n}$ are denoised by employing the band-pass FIR filter. It improves the expected signal SNR (“Signal to Noise Ratio”) and results in an increased classification precision. An example of the filtered version of signal for the (RBBB) class is shown in Fig. 3-a. The de-noised signal ${\text{xf}}_{\text{n}}$ is down-sampled with a factor of ${\text{D}} = 3$. An example of the subsampled versions of signal for the RBBB class is shown in Fig. 3-b.

The decimated signal $xd_{n}$ is splitted into fixed length segments of 0.9 s durations. Onward each segment is decomposed into subbands via the application of a 3 stages wavelet decomposer. Computational Gain of the designed front-end processing chain over the fixed-rate counterpart is calculated by using Eq. (4) and Eq. (5). It results in 4-fold reduction in terms of count of additions and multiplications of the designed solution compared to the fixed-rate counterpart.

In next step, four statistical features are extracted from each subband. In this way each intended instance is presented by 16 parameters. The compression gain of the designed framework over the conventional equal is computed by using ${\text{R}}_{\text{COMP}} = \frac{{{\text{N}}_{\text{r}} }}{\text{P}}$. It results in 22.5-fold real-time compression gain of the proposed solution over the conventional equal.

Above results show that the devised solution outperforms the conventional equals in terms of processing efficiency and compression gain. However, due to the multirate processing feature it may lose its performance in terms of the precision. Therefore, the overall performance of the system is measured in terms of the accuracy of the classification process. The KNN classifier is employed with *k *= 5 configuration. Training and testing sets are made of 3 distinct classes. Total 450 instances are used. The 10-fold cross validation technique is used for all experiments. Classifier’s performance is quantified in terms of the accuracy and the specificity by using Eq. (6) and Eq. (7). The obtained results are summarized in Table 1. It shows that for the studied case, the obtained classification accuracies are high. The highest classification accuracy is obtained for the (WPW) class, 93.2%. The average classification accuracy of the designed framework is 91.87% with an average specificity of 0.947. It concludes that the suggested approach not only attains the outperformance in terms of compression gain and processing efficiency but it also secures an appropriate ECG arrhythmia classification precision.

**Table 1.** Classification performance for 3 class ECG dataset

ECG class	Classification accuracy (% age)	Specificity	Average accuracy (% age)	Average specificity
Normal (N)	90.3	0.935	91.87	0.947
RBBB	92.1	0.951		
WPW	93.2	0.956		

## Conclusion

In this paper a novel multirate ECG processing, subbands decomposition and classification framework is designed. The decomposed signal subband features are mined and in next step these are utilized by the mature k-Nearest Neighbor (KNN) based classifier for an effective arrhythmia diagnosis. The multirate feature diminishes the system processing load. It is shown that because of the multirate feature the system has attained the 4 folds diminishing in the count of processing load as compared to the conventional equals. Additionally, the features extraction process has induced 22.5 times compression gain in the system. It also assures a same factor of processing load diminishing at the post classification stage. The overall performance of the system is quantified in terms of the accuracy of the classification process. For the studied case the designed framework has attained the highest classification accuracy of 93.2% and specificity of 0.956. It assures that the devised solution is a potential candidate to be embedded in contemporary automatic and mobile cardiac diseases diagnosis systems. A possible future direction of work is to adopt a model-based testing methodology for validating the proposed approach [15–18]. Integration and investigation of event-based processing modules [19–22] in this system is another prospect.
